# Association of Elevated Serum Aldosterone Concentrations in Pregnancy with Hypertension

**DOI:** 10.3390/biomedicines11112954

**Published:** 2023-11-01

**Authors:** Robin Shoemaker, Marko Poglitsch, Dolph Davis, Hong Huang, Aric Schadler, Neil Patel, Katherine Vignes, Aarthi Srinivasan, Cynthia Cockerham, John A. Bauer, John M. O’Brien

**Affiliations:** 1 Department of Dietetics and Human Nutrition, University of Kentucky, Lexington, KY 40506, USA; 2 Attoquant Diagnostics GmbH, 1110 Vienna, Austria; 3 Department of Pediatrics, University of Kentucky, Lexington, KY 40536, USA; 4 Department of Obstetrics and Gynecology, University of Kentucky, Lexington, KY 40536, USA

**Keywords:** hypertension, aldosteronism, renin, pregnancy, RAAS profiling, mass spectrometry, biomarkers

## Abstract

Emerging evidence indicates a previously unrecognized, clinically relevant spectrum of abnormal aldosterone secretion associated with hypertension severity. It is not known whether excess aldosterone secretion contributes to hypertension during pregnancy. We quantified aldosterone concentrations and angiotensin peptides in serum (using liquid chromatography with tandem mass spectrometry) in a cohort of 128 pregnant women recruited from a high-risk obstetrics clinic and followed prospectively for the development of gestational hypertension, pre-eclampsia, superimposed pre-eclampsia, chronic hypertension, or remaining normotensive. The cohort was grouped by quartile of aldosterone concentration in serum measured in the first trimester, and blood pressure, angiotensin peptides, and hypertension outcomes compared across the four quartiles. Blood pressures and body mass index were greatest in the top and bottom quartiles, with the top quartile having the highest blood pressure throughout pregnancy. Further stratification of the top quartile based on increasing (13 patients) or decreasing (19 patients) renin activity over gestation revealed that the latter group was characterized by the highest prevalence of chronic hypertension, use of anti-hypertensive agents, pre-term birth, and intrauterine growth restriction. Serum aldosterone concentrations greater than 704 pmol/L, the 75th percentile defined within the cohort, were evident across all categories of hypertension in pregnancy, including normotensive. These findings suggest that aldosterone excess may underlie the development of hypertension in pregnancy in a significant subpopulation of individuals.

## 1. Introduction

An estimated 15.3% of pregnancies are affected by hypertensive disorders of pregnancy (HDP) [1,2], and the prevalence is rising [3]. HDP and related adverse outcomes are major contributors to maternal and fetal morbidity and mortality [3]. HDP are a group of disorders collectively characterized by elevated blood pressure during pregnancy with multiple (mostly unknown) underlying causes, including pre-existing hypertension during pregnancy (chronic hypertension), gestational hypertension, and pre-eclampsia. Rational strategies for identifying subtypes of HDP could improve the management and/or treatment of hypertension during pregnancy.

Aldosterone plays a key role in the physiological regulation of fluid homeostasis, including the expansion of blood volume to meet the demands of pregnancy, and modulates other physiological processes in the heart and blood vessels [4]. Aldosterone excess is considered pathological, contributing to vascular injury and endothelial dysfunction, as well as cardiac inflammation and hypertrophy [5]. Aldosteronism is a group of conditions characterized by abnormal production of aldosterone that can be primary (caused by adrenal adenoma, uni- or bilateral adrenal hyperplasia, or, rarer, familial hyperaldosteronism, an inherited condition), with aldosterone production inappropriately high relative to sodium status, renin activity, and potassium levels, and non-suppressible in response to volume or sodium loading [6], or secondary (attributed to conditions such as renal artery stenosis [7], or reflecting fluid or electrolyte depletion [8]), which may include activation of the renin–angiotensin system. Detection of uncontrolled aldosterone secretion in individuals is important because this condition is linked with a higher incidence of target organ damage and cardiovascular events than age- and sex-matched patients with essential hypertension with the same degree of blood pressure elevation; treatment with mineralocorticoid receptor antagonists improves outcomes for these patients [8]. Emerging studies indicate a high prevalence of aldosteronism (multiple etiologies) among adults (perhaps up to 12% of the US population), especially among patients with severe hypertension [9,10]. Given the high rates of hypertension in reproductive-age women [11], it stands to reason that unrecognized aldosteronism may contribute to hypertension during pregnancy in a subpopulation of individuals, but there are little published data about this condition in pregnancy.

In a healthy pregnancy, plasma renin activity and aldosterone concentrations increase to accommodate blood volume expansion and the development of the placental-fetal unit [12,13]. Previous studies by our lab and others have reported that gestational hypertension and/or pre-eclampsia is accompanied by attenuated renin activity and lower aldosterone concentrations compared to normotensive pregnancies [14,15,16,17], presumably reflecting a response to increased systemic vascular resistance. Few studies have investigated the condition of aldosterone excess in pregnancy; the literature comprises only a handful of retrospective studies and case reports of pregnant patients with confirmed aldosteronism. Collectively, pregnancies with confirmed primary aldosteronism are indicated to be high-risk, with a highly variable disease course, and associated with high rates of maternal and fetal complications, such as pre-eclampsia and intrauterine growth restriction [18,19,20]. Outside of pregnancy, primary aldosteronism is characterized by an elevated aldosterone-to-renin ratio in blood that exceeds a defined cut-off value [8]; clear criteria for detection of aldosteronism where the renin–angiotensin system is also activated are not defined. Detection of any form of aldosteronism during pregnancy is complicated by the fact that renin activity and aldosterone concentrations increase in normal pregnancy, and reference values for these biomarkers in normotensive pregnancy have not been defined. Further, accumulating evidence in non-pregnant individuals indicates that a previously unrecognized and clinically relevant spectrum of abnormal aldosterone production may exist [10]; this has not been examined at all in pregnancy. 

To being to address these gaps, we used a previously described [17], liquid chromatography with tandem mass spectrometry (LC-MS/MS)-based method to quantify angiotensin peptides and aldosterone concentrations in serum in a cohort of pregnant women followed prospectively for the development of HDP (remaining normotensive, chronic hypertension, gestational hypertension, pre-eclampsia, superimposed pre-eclampsia on chronic hypertension). The aims of our study were to define associations among aldosterone concentrations in serum with blood pressure and hypertension outcomes in pregnancy, and to characterize activity of the RAAS in those with the highest levels of aldosterone. Recognition of the scope of potential abnormal aldosterone production in pregnancy could better define the prevalence of this condition, and inform the understanding of the pathogenesis and treatment of hypertension during pregnancy.

## 2. Materials and Methods

### 2.1. Study Population

This was a secondary analysis of 128 pregnant individuals enrolled in an on-going study at the University of Kentucky. All subjects gave informed consent to participate in this study, which was approved on 17 February 2019 by the University of Kentucky Institutional Review Board (#47841), and all study procedures were performed in accordance with relevant guidelines and regulations. Patients with moderate to high clinical risk factors for pre-eclampsia (presence of more than one of the following clinical risk factors: history of preterm pre-eclampsia, type 1 or 2 diabetes, renal or autoimmune disease; or more than two of the following clinical risk factors: nulliparity, obesity (body mass index > 30), family history of pre-eclampsia, Black race, lower income, age 35 or older, previous pregnancy with small birth weight or adverse outcome, or in vitro conception) [21] were recruited from high-risk obstetrics clinics in the Division of Maternal Fetal Medicine. The age range was between 18 and 45 years. Patients were excluded if they had multifetal gestation or anomalous fetus, allergy to aspirin, gastrointestinal bleeding, severe peptic ulcer or liver dysfunction, or were taking anticoagulant medications. 

### 2.2. Data Collection

Study clinicians (who are also providers for the patients in the cohort) in the Maternal Fetal Medicine unit identified potential patients, performed consenting, and enrolled participants at the time of first trimester screening (routine pre-natal visit or ultrasound appointment). Data were collected by study clinicians as part of routine clinical care over the course of pregnancy, and patients were treated according to clinical guidelines. Demographic information was collected upon enrollment. Clinical data and maternal blood were collected from routine visits in the first (12 weeks) and third (28 weeks) trimesters of pregnancy. Patients were followed prospectively for the development of pre-eclampsia, gestational hypertension, and chronic hypertension. Clinical data and outcomes were obtained from electronic medical records by a trained clinical coordinator, and input into a REDCap database by study personnel.

Systolic blood pressure (SBP) and diastolic blood pressure (DBP) were measured following the American Heart Association Guidelines [22], and were determined as the average of two consecutive measurements per arm assessed in a seated, resting position by one observer. Maternal blood was collected during routine prenatal laboratory evaluations in the first trimester (mean of 12 gestational weeks) and in the third trimester (mean of 28 gestational weeks). Blood was drawn by trained phlebotomists from patients in a seated position and transferred into red-top serum tubes. Study personnel transferred the whole blood samples to the laboratory for processing and storage. Whole blood was allowed to rest at room temperature for 60 min, followed by centrifugation, transferred into 200 uL aliquots, and barcoded by study personnel. Samples were stored at −80 °C until analysis for angiotensin peptides and aldosterone.

Pregnancy outcomes were determined during pregnancy and after delivery by trained clinical staff following guidelines published by the American College of Gynecologists [23]. Gestational hypertension was diagnosed as either SBP or DBP greater than 140 or 90 mmHg, respectively, after 20 weeks gestation in women with previously normal blood pressure, and chronic hypertension was determined by hypertension pre-dating pregnancy, or presenting before the 20th week of gestation. Pre-eclampsia was diagnosed with either SBP or DBP greater than 140 or 90 mmHg, respectively, after 20 weeks gestation, and one of the following signs indicating proteinuria, renal or kidney damage, low platelets, edema, or other neurologic features; pre-eclampsia in individuals with chronic hypertension was described as super-imposed pre-eclampsia. The following outcomes were also recorded: gestational diabetes, intrauterine growth restriction (defined as fetal weight below the 10th percentile), and pre-term birth (<37 weeks).

### 2.3. Quantification of Components of the RAAS: Angiotensin Peptides, Aldosterone, and Equilibrium-Based Biomarkers

Quantification of angiotensin I, angiotensin II, and aldosterone from serum samples of study patients was performed using LC-MS/MS-based methodology that has been previously described [17,24,25]. Serum samples were incubated for one hour at 37 °C to generate a controlled ex vivo equilibration, followed by stabilization through addition of an enzyme inhibitor cocktail (i.e., equilibrium analysis). Samples, calibrators, and quality controls were spiked with stable isotope-labeled internal standards (200 pg/mL for each angiotensin metabolite, used to correct for peptide recovery), subjected to C-18-based solid-phase extraction, followed by ultra-high-pressure liquid chromatography-based separation on a reversed-phase analytical column (Acquity UPLC C18, Waters, Milford, MA, USA) operating in line with a Xevo TQ-S triple quadruple mass spectrometer (Waters, Milford, MA, USA). Multiple reaction monitoring mode was used for generation of total ion chromatograms obtained from sums of quantifier transitions for each analyte, with previously optimized conditions for sensitivity, specificity, and signal-to-noise ratio. Integrated signals were used to determine analyte concentrations based on via linear calibration (Software: MassLynx/Target/Lynx, Version 4.2, Waters, Milford, MA, USA). Calculated levels of quality controls and calibrators were used to evaluate batch performance. The lower limits of quantification for angiotensin I and angiotensin II were 5 and 4 pmol/L, respectively, and 13 pmol/L for aldosterone.

Biomarkers of plasma renin activity and the aldosterone-to-Ang II ratio (AA2-R) were calculated from equilibrium concentrations of angiotensin peptides I and II and aldosterone [17,26,27]. The plasma renin activity surrogate (PRA-S) is the calculated sum of the equilibrium concentrations of angiotensins I and I ((eqAngiotensin I) + (eqAngiotensin II), pmol/L), and the AA2-R is the ratio of the concentrations of aldosterone to angiotensin II ((Aldosterone)/(eqAngiotensin II), (pmol/L)/(pmol/L)). These biomarkers are well-published in the literature as correlating with clinical characteristics and/or prediction outcomes in studies of hypertension [28,29], aldosteronism [26,27], heart failure [24,30], and more [31].

### 2.4. Statistical Analysis

Statistical analyses were performed via GraphPad Prism version 9.5.1 (San Diego, CA, USA). All data were assessed for normality, and the appropriate parametric/nonparametric tests were used. A *p*-value less than 0.05 was considered statistically significant. Paired clinical and biochemical data from the first and third trimester were obtained for 128 pregnant women. RAAS components were analyzed within-group for change over time (first trimester to third trimester), using Wilcoxon matched pairs signed-rank tests. Between-group differences in categorical variables (demographics) were analyzed using chi-square or Fisher’s exact test. Between-group differences were analyzed using one-way analysis of variance (ANOVA) followed by Dunnett’s multiple comparisons test for continuous, parametric variables (blood pressure, age, body mass index, gestational age at delivery) for more than two groups or *t*-tests for between-group comparison of two groups. The Kruskal–Wallis test followed by Dunn’s multiple comparisons test was used for between-group differences for non-parametric variables (RAAS components).

## 3. Results

### 3.1. Description of the Population

**Figure 1:**
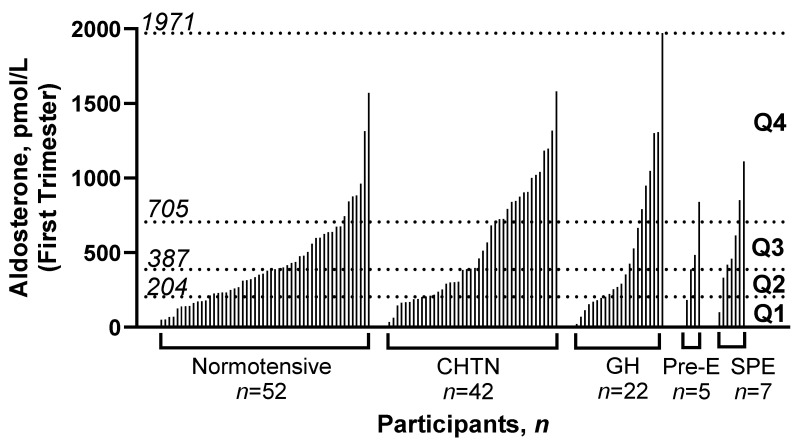
Distribution of serum aldosterone concentrations in the first trimester of pregnancy in the whole cohort. Serum aldosterone concentrations in the first trimester of pregnancy for each individual patient, ordered from lowest to highest, and grouped by hypertension outcome. Dotted lines indicate the value of aldosterone in pmol/L corresponding to the 25th percentile, median, 75th percentile, and maximum, for the 128 participants in the cohort. Q1: quartile one; Q2: quartile two; Q3: quartile three; Q4: quartile four; CHTN: chronic hypertension; GH: gestational hypertension; Pre-E: pre-eclampsia; SPE: superimposed pre-eclampsia.

The study population included 128 pregnant women, where 52 remained normotensive throughout pregnancy, 42 were determined to have chronic hypertension, 22 developed gestational hypertension, 5 developed pre-eclampsia, and 7 with chronic hypertension developed pre-eclampsia (superimposed pre-eclampsia). In Figure 1, the first trimester serum aldosterone concentration from each patient in the cohort is ordered from lowest to highest within each outcome. There is currently no defined reference range for normal aldosterone concentrations in blood in pregnancy, so aldosterone excess in this study was defined as a first trimester aldosterone concentration above the 75th percentile of the cohort. The cohort was divided into four groups of 32 patients each, based on quartile of aldosterone concentration in serum measured in the first trimester of pregnancy, and comparisons made across quartiles. The aldosterone value for the 25th percentile, median, 75th percentile, and maximum are indicated on the y-axis, indicating the range for each quartile, Q1–Q4, respectively.

Demographics and clinical characteristics of each quartile are displayed in Table 1. The cohort was primarily Caucasian, with Black, Hispanic, and mixed-race individuals distributed fairly equally across quartiles, with the exception of Q1, where there were no Hispanics and more Black patients compared to the other groups. The mean age of the whole cohort was 29.5 years, and did not differ widely across quartiles. The mean body mass index of the whole cohort was 32.9 kg/m^2^, likely reflecting the high-risk patient population, and there was a trend of greater body mass index in Q1 and Q4. Q1 had the most type 1 diabetics, but otherwise diabetes (and gestational diabetes) was distributed fairly equally among the four quartiles.

There was a notable difference in blood pressure across the quartiles, where SBP and DBP were significantly elevated in Q4 in both the first and third trimesters (Table 1), with Q1 having the next highest mean blood pressure compared to Q2 and Q3. Q4 contained the most patients with chronic hypertension and the fewest normotensive pregnancies compared to the other quartiles, and the most patients taking labetalol or nifedipine for blood pressure control (Table 1). Previous studies of pregnancy reported an association of low activity of the RAAS with development of HDP [15,17]. In the current study, gestational hypertension was most prevalent in Q1 and Q4 (combined prevalence of 21.8%) compared to Q2 and Q3 (combined prevalence of 15.6%), but pre-eclampsia was evident across all four quartiles. Q1 had a slightly greater number of patients with previous pre-eclampsia. Pre-term birth was the most prevalent in Q1 and Q4 (combined prevalence of 25%) compared to Q2 and Q3 (combined prevalence of 10.9%). IUGR was reported only in patients in Q1 and Q4 (combined prevalence of 10.9%), and not in Q2 or Q3. Gestational age at delivery and infant birth weight trended towards lower in Q1 and Q4, but there was no significant difference between groups.

### 3.2. Biochemical Analysis of the Serum RAAS in Pregnancy Grouped by Quartile of First Trimester Aldosterone Concentrations in Serum

**Figure 2:**
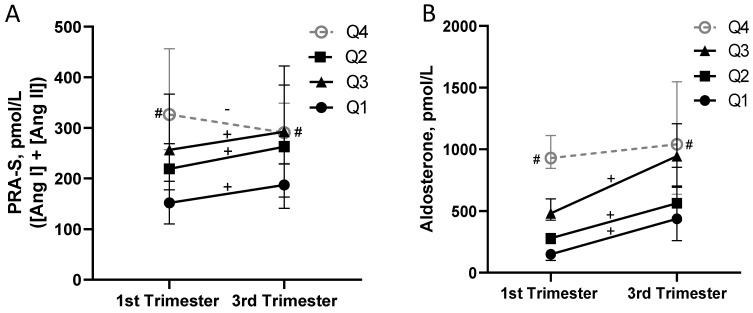
Activation of the RAAS in pregnancy is attenuated in the quartile (Q) with the highest concentrations of aldosterone in serum. (**A**) PRA-S, surrogate for plasma activity, calculated from the sum of the equilibrium concentrations of angiotensin I ([Ang I]) and angiotensin II ([Ang II]). #, *p* < 0.05 vs. Q3, *p* < 0.01 vs. Q2, and *p* < 0.001 vs. Q1 in the first trimester, and *p* < 0.05 vs. Q1 in the third trimester analyzed using the Kruskal–Wallis test followed by Dunn’s multiple comparisons test; + or −, *p* < 0.05 within-group increase or decrease in angiotensin I, angiotensin II, and/or PRA-S from first to third trimester analyzed using Wilcoxon matched pairs signed-rank tests. (**B**) Aldosterone concentrations. #, *p* < 0.01 vs. Q3, *p* < 0.001 vs. Q2, and *p* < 0.0001 vs. Q1 in the first trimester, and *p* < 0.001 vs. Q1 in the third trimester analyzed using the Kruskal–Wallis test followed by Dunn’s multiple comparisons test; +, *p* < 0.0001 positive change over gestation within group analyzed using Wilcoxon matched pairs signed-rank tests. Data are median with 95th confidence interval in 128 patients with 32 patients in each quartile.

We investigated activity of the RAAS in serum samples over gestation across the four quartiles, and the median values and interquartile range for all measured components of the RAAS are displayed in Table 2. In the first trimester, the median concentrations of aldosterone in each group progressively increased across quartiles, and there was a six-fold difference between the median values of Q1 and Q4 (Figure 2B). This trend corresponded with concentrations of the upstream components, angiotensin I and angiotensin II (and correspondingly, the calculated biomarker PRA-S, Figure 2A, Table 2), where these values progressively increased across quartiles, but only a two-fold difference in the respective values between Q1 and Q4 was observed. The aldosterone-to-angiotensin II ratio was increased four-fold between Q4 and Q1 in the first trimester (Table 2). The median and interquartile range for measured components of the RAAS in the first and third trimester in the whole cohort of 128 patients are included as Supplementary Table S1.

Previous studies demonstrated increased activity of PRA-S and aldosterone concentrations over gestation in normotensive pregnancies [12,17]. In the current study, the concentrations of angiotensin I and aldosterone (and a trend for PRA-S) significantly increased from the first to the third trimester in Q1, Q2, and Q3 (Table 2, Figure 2A,B. In contrast, the concentrations of angiotensin I and aldosterone did not increase over gestation in the Q4 group (Table 2; Figure 2A,B). In fact, there was a significant decrease over gestation in the concentrations of angiotensin II in the Q4 group (*p* < 0.05), and this was reflected by a reduction in the PRA-S in the third vs. first trimester in this group only (*p* = 0.120; Table 2, Figure 2A).

### 3.3. Association of Declining Renin Activity over Gestation with Blood Pressure in Patients with the Highest Concentrations of Serum Aldosterone

**Figure 3:**
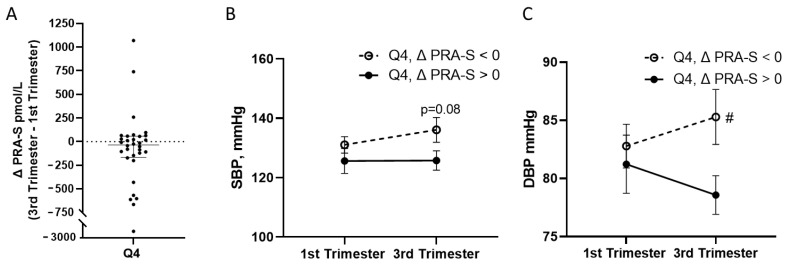
Systolic and diastolic blood pressure in in the quartile with the highest aldosterone concentrations in serum stratified by increasing or decreasing PRA-S from the first to third trimester. (**A**) ΔPRA-S, the change in the PRA-S (surrogate biomarker for plasma renin activity, calculated from sum of the equilibrium concentrations of angiotensin I and angiotensin II) value from the first to the third trimester: [PRA-S, third trimester]—[PRA-S, first trimester], in quartile four (Q4). Patients were stratified by increasing (13 patients) or decreasing (19 patients) ΔPRA-S. (**B**) Systolic blood pressure (SBP) and (**C**) diastolic blood pressure (DBP) in patients in Q4 with increasing vs. decreasing PRA-S. Data are median with 95th confidence interval. #, *p* < 0.05 between groups, analyzed using paired *t*-test.

Outside of pregnancy, aldosterone excess is often accompanied by suppressed renin activity [8], but there are no criteria to define suppressed renin activity in pregnancy. We recently demonstrated that declining PRA-S over gestation was linked to development of hypertension [17], so we performed a subanalysis of patients in Q4 based on whether PRA-S increased or decreased from the first to the third trimester (ΔPRA-S = [PRA-S, third trimester]—[PRA-S, first trimester]). Of the 32 patients in the Q4 group, there were 13 patients with increasing PRA-S, and 19 patients with decreasing PRA-S (Figure 3A). The SBP and DBP in the Q4 for these groups are depicted in Figure 3 and Table 3. In patients with high concentrations of aldosterone in the first trimester (Q4), decreasing PRA-S over gestation was associated with elevated SBP (*p* = 0.08; Figure 3B) and DBP (*p* < 0.01, Figure 3C) in the third trimester, compared to those where PRA-S increased.

For comparison, a similar analysis was performed to examine the association of declining PRA-S over gestation with third trimester blood pressure in the remaining quartiles, Q1 + Q2 + Q3 (sum of patients with first trimester aldosterone concentrations below the 75th percentile). There were 37 patients in Q1 + Q2 + Q3 where PRA-S decreased (including one patient with ΔPRA-S = 0), and 59 patients in quartiles Q1 + Q2 + Q3 where PRA-S increased over gestation. SBP and DBP for these groups are lower than those reported for Q4 with declining PRA-S (Table 3).

In addition to elevated blood pressure, the group in the highest quartile for first trimester aldosterone concentration with declining PRA-S over pregnancy had the highest prevalence of chronic hypertension, the most patients taking antihypertensive agents for blood pressure control, a high incidence of pre-term birth, and the most cases of IUGR (Table 3). There was not an apparent association among gestational hypertension and elevated aldosterone concentrations or declining renin activity. Interestingly, there were no cases of pre-eclampsia in the Q4 group with increasing PRA-S, and pre-eclampsia was most prevalent in the combined two groups were PRA-S decreased over gestation (Table 3). Within Q4, there were notably more pre-term birth cases with decreasing PRA-S, and the Incidence of pre-term birth was overall greater with declining renin (Table 3).

## 4. Discussion

A recent study by Brown et al. across four academic medical centers reported a previously unrecognized high prevalence of primary aldosteronism present across all categories of hypertension, including normotensive individuals [10]. These data suggest aldosteronism might underlie the development of HDP in a subpopulation of pregnant individuals, but few studies have investigated pathological aldosteronism in pregnancy. We quantified concentrations of aldosterone and other upstream components of the RAAS in a cohort of 128 pregnant women with risk factors for HDP to determine whether high aldosterone concentrations in serum in the first trimester (defined within-study as values greater than the 75th percentile of the cohort) were associated with elevated blood pressure and/or hypertension outcomes in pregnancy. The main findings from this study are: (1) Aldosterone concentrations in serum greater than 704 pmol/L (the 75th percentile within cohort) were evident across all categories of hypertension in pregnancy, including normotensive; (2) systolic and diastolic blood pressures were highest in the top quartile of aldosterone concentration compared to the remaining quartiles, (3) especially when accompanied by declining renin activity, and this group was characterized by a high prevalence of chronic hypertension, use of anti-hypertensive agents, pre-term birth, and slightly higher prevalence of IUGR. The lowest quartile of aldosterone concentrations (less than 204 pmol/L; below the 25th percentile of the cohort) also had high-risk characteristics, such as elevated blood pressure, higher prevalence of type 1 diabetes, pre-term birth, and development of gestational hypertension. Taken together, these findings agree with those of previous studies that demonstrated lower renin activity and aldosterone levels in hypertensive pregnancies [14,15,16,32,33], and add to the literature evidence of a significant portion of pregnant women in our predominately high-risk cohort with very high concentrations of aldosterone in serum, which was associated with pre-pregnancy (chronic) hypertension and elevated blood pressure during pregnancy.

While it has been known for decades that pregnancies that develop gestational hypertension or pre-eclampsia have reduced activity of the RAAS compared to normotensive pregnancies [32,34], studies have not examined associations among high aldosterone levels and blood pressure in HDP. One reason for this is that elevated aldosterone concentrations are known to be a normal and necessary feature of healthy pregnancy: e.g., maternal aldosterone secretion was required for plasma volume expansion in pregnant ewes [35]; and aldosterone deficiency reduced placental function, litter size, and pup weight in mice [36]. The concept of aldosterone as a mediator of cardiovascular disease has emerged more recently, where attributes of modern lifestyles (physical inactivity, chronic stress, and energy overabundance) have led to an increase in the number of individuals with elevated secretion of aldosterone and/or activation of mineralocorticoid receptors with adverse effects on vascular function [4]. Females may be biologically primed for aldosterone secretion [37], which may have provided an evolutionary advantage for successful reproduction, but the recent literature indicates that females may be especially susceptible to cardiac and vascular dysfunction mediated by elevated angiotensin II [38] or aldosterone levels [39,40,41]. Being overweight and obesity are also associated with increased aldosterone production [42]. Increasing rates of obesity in pregnant and reproductive-age women may have resulted in the relatively recent emergence of a subpopulation of individuals with abnormally elevated secretion of during pregnancy.

A limitation to the study of aldosteronism during pregnancy is the absence of thresholds or ranges of concentrations of serum aldosterone considered to be normal vs. abnormal in pregnancy. Sanga et al. aimed to establish reference ranges for plasma aldosterone concentrations (in ng/dL) and direct renin concentration (in mU/L) in pregnancy, where values were derived from a historical study of 18 patients followed weekly throughout pregnancy and derived using immunoassay-based detection methods [43]. Mean plasma aldosterone concentrations were approximately 20 ng/dL (or converted to 555 pmol/L) in normal, healthy pregnancy at the 12th gestational week, and approximately 45 ng/dL (or converted to 1250 pmol/L) at the 28th gestational week [20]. We report here median and interquartile ranges of aldosterone concentrations in a cohort of 128 high-risk pregnant patients (a considerable sample size, comparatively [44,45,46]) at gestational weeks 12 (387 pmol/L, 204–705 pmol/L) and 28 (724 pmol/L, 376–1197 pmol/L) of pregnancy quantified via LC-MS/MS (the gold standard for determination of aldosterone in clinical assays; aldosterone concentrations derived from immunoassay-based methods are typically higher and more variable that when derived via mass spectrometry-based methods [47]). For reference, aldosterone concentrations outside of pregnancy measured using LC-MS/MS range between 100–300 pmol/L [47], and the median and interquartile range for aldosterone concentrations in 33 non-pregnant patients with confirmed aldosteronism were 392 pmol/L and 331 to 468 pmol/L [26].

There is not an internationally accepted, standardized methodology for quantification of renin activity. Plasma renin activity is the most sensitive and accurate method for assessing renin activity, especially in the clinically relevant low ranges, but direct renin concentration is often used because measurement is more convenient [48]. Mean direct renin concentrations at gestational weeks 12 and 28 reported by Sanga et al. from 18 patients in the literature were approximately 35 mU/L and 42 mU/L, respectively [20]. Comparatively, the median and interquartile range of direct renin concentration in 33 non-pregnant patients with confirmed aldosteronism were 3.8 mU/L and 2.9 to 7.5 mU/L, compared to the mean and interquartile range of 20.3 mU/L and 10.3 to 33 mU/L in 77 non-pregnant patients with essential hypertension [26]. While there is not a meaningful conversion rate between direct renin concentration and plasma renin activity, the latter study of non-pregnant patients also quantified PRA-S using the same methodology as the current study, and the median and interquartile range for 33 patients with confirmed aldosterone were 40 pmol/L and 18–58 pmol/L, compared to 165 pmol/L and 80–328 pmol/L in the 77 patients with essential hypertension. The median and interquartile ranges for PRA-S were reported in our study of pregnancy were comparatively higher (246 pmol/L and 153–353 pmol/L in the first trimester, and 264 pmol/L and 175–397 pmol/L in the third trimester). Results from our study confirm that PRA-S and aldosterone concentrations are elevated in pregnant compared to non-pregnant patients and add to the literature reference values for aldosterone and PRA-S concentrations, as well as concentrations of angiotensin I and angiotensin II (and other calculated biomarkers of RAAS activity) at equilibrium, measured using LC-MS/MS and reported in pmol/L, in the first and third trimesters of pregnancy in 128 patients (Supplementary Table S1).

The literature describing aldosteronism in pregnancy mostly comprises individual case reports, small retrospective studies based on medical chart review, and narrative reviews. Sanga et al. recently published the most comprehensive-to-date systematic review describing features of 83 cases available in the literature of patients with confirmed primary aldosteronism who underwent pregnancy. There were 56 cases of sporadic primary aldosteronism (uni- or bilateral hyperplasia) and 27 cases of familial hyperaldosteronism type 1 (inappropriate production of aldosterone by adrenocorticotropic hormone). In the latter, most cases were diagnosed before pregnancy, blood pressure was effectively managed, and there were fewer poor outcomes. Among the unilateral and bilateral hyperplasia cases, approximately 60% were characterized by known existence of pre-pregnancy hypertension, but less than half had a confirmed diagnosis of aldosteronism before pregnancy, and there was a complication rate of 50% in previous pregnancies. Of the 83 cases reviewed, 61% had complications in the current pregnancy, 46% had pre-term birth due to pre-eclampsia and/or fetal distress, 22% of the fetuses had intrauterine growth restriction, 7% of the neonates needed the intensive care unit, and 7% died.

Vidyasagar et al. recently described clinical characteristics and aldosterone concentrations (measured via LC-MS/MS) and direct renin concentration (via chemiluminescent immunoassay) in nine pregnant women with confirmed primary aldosteronism and 33 pregnant women with chronic hypertension via retrospective chart analysis [18]. In the nine cases of confirmed aldosteronism, 50% experienced pre-term delivery due to uncontrolled blood pressure, 37.5% had pre-eclampsia (compared to 15% in the chronic hypertension group), and there were two small for gestational age neonates, and two NICU admissions. The biochemical analysis of the RAAS in the nine patients with primary aldosteronism was performed in different weeks of gestation, ranging from 7–36 gestational weeks (mean of 28), and the mean and range of aldosterone values were 1038 pmol/L and 378–1870 pmol/L. All nine patients with confirmed aldosteronism had a direct renin concentration less than 20 mU/L, and this was significantly lower compared to the chronic hypertension patients. Aldosterone concentrations were comparatively similar, and direct renin concentrations were slightly lower in the nine patients with confirmed aldosterone compared to those reported by Sanga et al. at gestational week 28, but both biomarkers were elevated compared to non-pregnant patients with and without confirmed aldosteronism as described above. Results from our study are comparable, where the median and interquartile range of aldosterone concentrations at week 28 in the Q4 group were 1041 pmol/L and 586–1742 pmol/L, and in patients with declining renin activity over gestation, similar clinical characteristics were observed to those described in aldosteronism patients with lower direct renin concentrations.

A limitation to our study is that it did not include testing for, or confirmation of, aldosteronism as primary or secondary. However, several insights can be gleaned from our study when considered in context with the characteristics described in the preceding studies [18,20] and prevalence of aldosteronism reported by Brown et al. in the four-site study of 1015 patients receiving care for the treatment of hypertension [10]. The latter study demonstrated rates of 11–22% of renin-independent (primary) aldosteronism, which increased over the categories of normotensive, untreated stage 1 hypertension, untreated stage 2 hypertension, and treated resistant hypertension; the rates increased among a subset with suppressed renin activity, especially in the treated resistant group (51.6% prevalence of confirmed aldosteronism). In our study, the aldosterone excess group was defined as the top 25% of aldosterone concentrations, and when patients within this group were selected for declining renin activity over gestation (considering that suppressed renin activity is a feature of primary aldosteronism outside of pregnancy), there were 19 out of 128 patients, about 15%, which is consistent with current estimates of primary aldosteronism in the population. Similar to those reviewed above [20], 68% of these patients in our study had chronic hypertension; mean blood pressure was elevated despite more antihypertensive use, there were three intrauterine growth restriction cases (15% prevalence, which was greater than the other groups), and the highest prevalence (31.6%) of pre-term birth was noted.

The clinical utility of the aldosterone-to-renin ratio for detection of primary aldosteronism in pregnancy is unclear. First, the timing of determination of aldosterone and renin for detection of aldosteronism matters during pregnancy; there is an upward shift in the aldosterone and renin ranges compared to non-pregnant states [44], which rises over gestation, but the rise in aldosterone concentrations is proportionally greater than the rise in renin activity over the course of pregnancy (reflected in our study by a doubling in the aldosterone-to-angiotensin II ratio from the first to third trimester of pregnancy in the whole cohort). Second, outside of pregnancy, the elevated aldosterone-to-renin ratio in patients with primary aldosteronism results from renin-independent secretion of aldosterone leading to suppression of renin (as part of a regulatory negative feedback loop) [8]. Whether mechanisms of the normal RAAS response to pregnancy (increased renin activity and aldosterone secretion) are altered in patients with pre-existing aldosteronism (of multiple etiologies) is simply not known. We did note a small number of patients in Q4 that also had very elevated values of PRA-S, which could also be attributed to secondary aldosteronism resulting from renovascular hypertension [7], which has been reported to present as pre-eclampsia [49]. Moreover, abnormalities in the mineralocorticoid effector mechanisms (such as reduced mineralocorticoid receptor number in mononuclear leukocytes in the absence of abnormal plasma aldosterone levels) have been reported as a potential contributor to the development of pre-eclampsia [50,51]. Further, highly elevated levels of progesterone in pregnancy may promote aldosterone production independently from the renin–angiotensin system [52] or even interfere with aldosterone signaling through the mineralocorticoid receptor [53]. Results from a recent trial, treatment for mild chronic hypertension during pregnancy, underscore the important of blood pressure control in pregnancy for the prevention of adverse outcomes [54], and patients with hypertension in our study were treated with labetalol (a beta blocker) or nifedipine (a calcium channel blocker in the dihydropyridine class), where the former can decrease and the latter can increase plasma renin activity [55], potentially confounding interpretation of RAAS in pregnancy. Likewise, the ratio of uric acid to creatinine in serum is associated with the development of pre-eclampsia [56]. While our study design did not include collection of these variables, it is important to note that hyperuricemia may lead to increased renin activity in some hypertensive individuals [57].

In the current study, our analysis was based on aldosterone concentrations measured in the first trimester, prior to the peak pregnancy-mediated response. In all quartiles, plasma renin activity (estimated using a surrogate value for plasma renin activity, PRA-S, calculated from the sum of the concentrations of angiotensin I and angiotensin II) was proportional to aldosterone concentrations in the first trimester, and was highest in the top quartile. However, only in the top quartile did median PRA-S values decrease from first to third trimester, and aldosterone concentrations did not significantly rise over gestation (in contrast to the other quartiles). These data suggest that renin activity and aldosterone secretion may increase initially in response to pregnancy in patients with excess aldosterone (values are much higher compared to non-pregnant with or without confirmed aldosteronism), and declining renin activity over gestation with prolonged secretion of high levels of aldosterone may be indicative of feedback suppression on renin as part of the pathology of aldosteronism. A limitation to our study is that we did not compare PRA-S values to traditional plasma renin activity assays, although previous studies outside of pregnancy reported a strong correlation among values derived via PRA-S and plasma renin activity [26,58]. Advantages of the LC-MS/MS-based methods used in our study are that multiple effectors are quantified in a single analytic run, calculated biomarkers (e.g., for PRA-S) are derived from directly measured concentrations of effectors at high sensitivity, and the reproducibility of mass spectrometry allows for comparison across multiple studies.

Additional limitations in our study to consider include a high prevalence of overweight and obesity in our cohort, where body fat percentage is linked to risk of aldosteronism [59]. Additionally, sodium or fluid status was not controlled in our study. Differences in sodium, renin, and aldosterone relationships in pregnancy hypertension vs. normotensive are not well understood [60,61,62]. Moreover, other hormones with important roles in fluid adaptation, such as vasopressin, have been associated with development of pre-eclampsia [63,64].

## 5. Conclusions

Results from our study agree with those of previous studies where lower renin activity and aldosterone concentrations were associated with pre-eclampsia/hypertension in pregnancy, and report new evidence of an association between very high concentrations of aldosterone in serum in the first trimester of pregnancy and a phenotype of elevated blood pressure. Further stratification by increasing or decreasing renin activity over gestation revealed a subset of patients with a higher prevalence of chronic hypertension, use of anti-hypertensive medications, pre-term birth, and slightly higher prevalence of IUGR. These findings suggest that aldosterone excess may underlie the development of hypertension in pregnancy in a significant subpopulation of individuals.

## Figures and Tables

**Table 1:** Patient demographics, clinical characteristics, and outcomes of pregnant women based on quartile of first trimester aldosterone concentration in serum.

Parameter	Q1	Q2	Q3	Q4
	*n* = 32	*n* = 32	*n* = 32	*n* = 32
*n* (%) or mean ± SD				
Race				
Caucasian	25 (78.1)	20 (62.5)	22 (68.8)	23 (71.9)
Black or African American	7 (21.9)	4 (12.5)	4 (12.5)	4 (12.5)
Hispanic	0	5 (15.6)	4 (12.5)	3 (9.4)
Asian	0	1 (3.1)	0	1 (3.1)
Mixed race/other	0	2 (6.3)	1 (3.1)	1 (3.1)
Primiparous	9 (28.1)	10 (31.3)	9 (28.1)	7 (21.9)
Previous pre-e	7 (21.9)	2 (6.3)	5 (15.6)	2 (6.3)
Type 1 diabetes	10 (31.3)	5 (15.6)	2 (6.3)	4 (12.5)
Type 2 diabetes	3 (9.4)	6 (18.8)	9 (9.4)	3 (9.4)
Gestational diabetes	4 (12.5)	4 (12.5)	2 (6.3)	5 (15.6)
Medications				
Aspirin	25 (78.1)	21 (65.6)	18 (56.3)	27 (84.4)
Labetalol or nifedipine	4 (12.5)	1 (3.13)	3 (9.4)	10 (31.3) ^@^
Anti-diabetic agents	14 (43.8)	10 (31.3)	7 (21.9)	9 (28.1)
Age, years	27 ± 4 ^#^	30 ± 6	31 ± 7	30 ± 6
BMI, kg/m^2^	35.0 ± 10.3	31.4 ± 7.8	31.5 ± 10.3	34.0 ± 10.4
1st trimester blood pressure				
SBP, mmHg	125 ± 13	119 ± 13	120 ± 15	129 ± 13 *
DBP, mmHg	79 ± 7	75 ± 7	77 ± 9	82 ± 4 **
3rd trimester BP				
SBP, mmHg	125 ± 12	122 ± 14	120 ± 14	132 ± 17 ^^^
DBP, mmHg	78 ± 6	76 ± 8	77 ± 9	83 ± 9 ^^^^
Gestational age at delivery, weeks	36.8 ± 2.3	38.1 ± 1.7	38.1 ± 1.7	37.1 ± 2.2
Infant birth weight, grams	3123 ± 711	3394 ± 550	3366 ± 559	3122 ± 776
Normotensive	12 (37.5)	16 (50.0)	17 (53.1)	7 (21.9)
Chronic HTN	9 (28.1)	10 (31.3)	7 (21.9)	16 (50.0) ^@^
Gestational HTN	8 (25.0)	5 (15.7)	3 (9.4)	6 (18.8)
Pre-eclampsia or SPE	3 (9.4)	1 (3.13)	5 (15.7)	3 (9.4)
Pre-term birth	9 (28.0)	2 (6.3)	5 (15.6)	7 (21.9)
IUGR	3 (9.4)	0	0	4 (12.5)

Abbreviations BMI: body mass index, first trimester; SBP: systolic blood pressure; DBP: diastolic blood pressure; HTN: hypertension; SPE: super-imposed pre-eclampsia; IUGR: intrauterine growth restriction. ^@^, *p* < 0.05 difference between groups using chi-square analysis; ^#^, *p* < 0.05 vs. Q3; *, *p* < 0.05, and **, *p* < 0.01 vs. Q2, Q3; ^^^, *p* < 0.05, and ^^^^, *p* < 0.01 vs. Q2, Q3 analyzed using one-way analysis of variance followed by Dunnett’s multiple comparisons test.

**Table 2:** Concentrations of equilibrium angiotensin peptides, aldosterone, and biomarkers determined by liquid chromatography with tandem mass spectrometry in the first and third trimesters of pregnancy by quartile of aldosterone.

Parameter	Q1	Q2	Q3	Q4
	*n* = 32	*n* = 32	*n* = 32	*n* = 32
Median (Interquartile Range)				
Aldosterone, pmol/L				
1st Trimester	149.8 (70.58–178.2)	279.9 (232.8–328.0)	482.1 (416.1–612.1)	928.6 (840.2–1194.0) ^#^
3rd Trimester	437.2 (247.4–802.8) ****	562.5 (336.8–1126) ****	944.8 (642.6–1359) ****	1041.0 (585.9–1742.0) ^#^
Angiotensin I, pmol/L				
1st Trimester	41.8 (25.3–67.6)	69.9 (49.9–92.1)	86.09 (59.1–125.9)	107.0 (89.85–161.9) ^#^
3rd Trimester	63.6 (45.1–131.7) ***	95.8 (55.7–148.7) *	121.6 (69.9–176.1) *	108.5 (75.25–157.3) ^#^
Angiotensin II, pmol/L				
1st Trimester	104.3 (71.33–160.1)	145.1 (110.9–189.4)	172.6 (140.3–266.9)	217.0 (169.8–350.3) ^#^
3rd Trimester	107.6 (70.21–180.2)	158.9 (90.97–281.9)	176.0 (132.2–277.0)	172.5 (122.8–280.6) *^,#^
PRA-S, pmol/L				
1st Trimester	151.8 (101.7–225.7)	219.1 (148.6–280.4)	256.8 (197.8–612.1)	326.3 (250.5–494.3) ^#^
3rd Trimester	187.3 (123.2–322.8) *	262.8 (139.5–422.9)	292.5 (221.9–466.0)	290.7 (205.6–456.3) ^#^
AA2-R, (pmol/L)/(pmol/L)				
1st Trimester	0.95 (0.71–1.62)	1.99 (1.41–2.45)	3.28 (2.10–4.29)	4.63 (2.58–6.84) ^#^
3rd Trimester	3.90 (2.3–7.0) ****	3.50 (2.43–6.70) ****	4.81 (3.20–7.72) ****	5.25 (3.22–9.79) *,^#^

Abbreviations: PRA-S: surrogate biomarker for plasma renin activity, calculated from sum of the equilibrium concentrations of angiotensin I ([Ang I]) and angiotensin II ([Ang II]); AA2-R: aldosterone-to-angiotensin II ratio. *, *p* < 0.05, ***, *p* < 0.001, ****, *p* < 0.0001 for third trimester vs. first trimester within group analyzed by Wilcoxon signed-rank test. Between-group analysis using the Kruskal–Wallis test followed by Dunn’s multiple comparisons test: Aldosterone: ^#^, *p* < 0.01 vs. Q3, *p* < 0.001 vs. Q2, and *p* < 0.0001 vs. Q1 in the first trimester, and *p* < 0.001 vs. Q1 in the third trimester. Angiotensin I: ^#^, *p* < 0.01 vs. Q2 in the first trimester, and *p* < 0.05 vs. Q1 in the third trimester. Angiotensin II: ^#^, *p* < 0.05 vs. Q3 in the first trimester, and *p* < 0.05 vs. Q1 in the third trimester. PRA-S: ^#^, *p* < 0.05 vs. Q3, *p* < 0.01 vs. Q2, and *p* < 0.001 vs. Q1 in the first trimester, and *p* < 0.05 vs. Q1 in the third trimester. AA2-R: ^#^, *p* < 0.001 vs. Q2 and *p* < 0.0001 vs. Q1 in the first trimester.

**Table 3:** Characteristics of patients based on increasing or decreasing renin activity over pregnancy, in the top aldosterone quartile compared to the sum of the remaining quartiles.

	Q4 (>75th Percentile)		Q1 + Q2 + Q3 (<75th Percentile)	
	ΔPRA-S < 0 *n* = 19	ΔPRA-S > 0 *n* = 13	ΔPRA-S < 0 *n* = 37	ΔPRA-S > 0 *n* = 59
Mean ± SD or *n* (%)				
BMI, kg/m^2^	34.5 ± 9.2	33.3 ± 12.3	33.9 ± 11.1	31.8 ± 8.5
SBP, mmHg	136 ± 18 ^#^	126 ± 12	126 ± 15	120 ± 11
DBP, mmHg	85 ± 10 ^#^	79 ± 6	78 ± 7	76 ± 8
Chronic hypertension	13 (68.4) ^^^	5 (38.5)	14 (38)	18 (30.5)
Antihypertensive agents	7 (36.8) ^^^	3 (23.1)	5 (13.5)	3 (5.1)
Pre-eclampsia	3 (15.8)	0	8 (21.6)	2 (3.4)
Gestational hypertension	3 (15.8)	3 (23.1)	8 (21.6)	10 (16.9)
Pre-term birth	6 (31.6) ^^^	1 (7.7)	10 (27.0)	6 (16.2)
IUGR	3 (15.8) ^^^	1 (7.7)	1 (2.7)	2 (3.4)

Abbreviations: ΔPRA-S: [PRA-S, third trimester]—[PRA-S, first trimester] (i.e., increasing or decreasing renin activity over gestation); BMI: body mass index, first trimester; SBP: systolic blood pressure in the third trimester; DBP: diastolic blood pressure in the third trimester; IUGR: intrauterine growth restriction. ^#^, *p* < 0.01 vs. Q1 + Q2 + Q3 with ΔPRA-S less than zero for SBP; ^#^, *p* < 0.01 vs. all other groups for DBP analyzed using one-way analysis of variance followed by Dunnett’s multiple comparisons test; ^^^, *p* < 0.05 difference between groups using Fisher’s exact test.

## Data Availability

Data may be made available by investigators upon reasonable request to the corresponding author: robin.shoemaker@uky.edu.

## Supplementary Materials

The following supporting information can be downloaded at: https://www.mdpi.com/article/10.3390/biomedicines11112954/s1, Table S1: Concentrations of RAAS components measured in a cohort of 128 patients in the first and third trimesters of pregnancy.
