# Supplementary material for: Association of Elevated Serum Aldosterone Concentrations in Pregnancy with Hypertension
Source: Biomedicines. 2023 Nov 1;11(11):2954. doi: 10.3390/biomedicines11112954 (PMC10669350; doi:10.3390/biomedicines11112954)
Supplement: Supplementary file 1 [file biomedicines-11-02954-s001.zip › biomedicines-2671912-supplementary.pdf]

Supplementary Table S1: Concentrations of RAAS components measured a cohort of 128 patients in the first and third trimesters of pregnancy.

RAAS Component	1st Trimester n=128	3rd Trimester n=128
Median (IQR)		
Aldosterone, pmol/L	387.2 (204.3 - 705.1)	724.8 (376.6 - 1197)
Angiotensin I, pmol/L	77.54 (47.31 - 112.1)	100.2 (58.90 - 151.8)
Angiotensin II, pmol/L	164.9 (101.4 - 227.9)	158.2 (105.7 - 243.8)
PRA-S, pmol/L	246.0 (152.9 - 353.1)	265.4 (174.6 - 397.0)
AA2-R, (pmol/L)/(pmol/L)	2.235 (1.317 - 4.150)	4.518 (2.663 - 7.308)
